# Transient ectopic expression of the histone demethylase JMJD3 accelerates the differentiation of human pluripotent stem cells

**DOI:** 10.1242/dev.139360

**Published:** 2016-10-15

**Authors:** Tomohiko Akiyama, Shunichi Wakabayashi, Atsumi Soma, Saeko Sato, Yuhki Nakatake, Mayumi Oda, Miyako Murakami, Miki Sakota, Nana Chikazawa-Nohtomi, Shigeru B. H. Ko, Minoru S. H. Ko

**Affiliations:** Department of Systems Medicine, Keio University School of Medicine, Tokyo 160, Japan

**Keywords:** Histone demethylase, JMJD3, KDM6B, Human pluripotent stem cells, Hepatocytes, Skeletal muscle

## Abstract

Harnessing epigenetic regulation is crucial for the efficient and proper differentiation of pluripotent stem cells (PSCs) into desired cell types. Histone H3 lysine 27 trimethylation (H3K27me3) functions as a barrier against cell differentiation through the suppression of developmental gene expression in PSCs. Here, we have generated human PSC (hPSC) lines in which genome-wide reduction of H3K27me3 can be induced by ectopic expression of the catalytic domain of the histone demethylase JMJD3 (called JMJD3c). We found that transient, forced demethylation of H3K27me3 alone triggers the upregulation of mesoendodermal genes, even when the culture conditions for the hPSCs are not changed. Furthermore, transient and forced expression of JMJD3c followed by the forced expression of lineage-defining transcription factors enabled the hPSCs to activate tissue-specific genes directly. We have also shown that the introduction of JMJD3c facilitates the differentiation of hPSCs into functional hepatic cells and skeletal muscle cells. These results suggest the utility of the direct manipulation of epigenomes for generating desired cell types from hPSCs for cell transplantation therapy and platforms for drug screenings.

## Abstract (precis)

**Summary:** Forced expression of the catalytic domain of JMJD3 together with lineage-defining transcription factors can dramatically accelerate the differentiation of human PSCs.

## INTRODUCTION

Pluripotent stem cells (PSCs), such as human embryonic stem cells (hESCs) (Thomson et al., 1998) and human induced pluripotent stem cells (hiPSCs) (Takahashi et al., 2007; Yu et al., 2007), have the capacity to differentiate into various cell types, accompanied by dynamic alterations in gene expression patterns (Drukker et al., 2012; Fathi et al., 2011; Norrman et al., 2013; Yang et al., 2013). hESCs/iPSCs maintain their undifferentiated state by expressing pluripotency-associated genes, such as *POU5F1*, *NANOG* and *SOX2*. hESC/iPSC differentiation involves the suppression of these pluripotency-associated genes and the activation of early developmental genes, followed by the activation of tissue lineage-defining genes.

Based on our current knowledge of developmental processes, *in vitro* culture conditions containing suitable growth factors or cytokines have been developed to activate the signaling pathways for hPSC differentiation towards specific cell lineages (Murry and Keller, 2008). However, in most cases, the low differentiation efficiency and the requirement for long-term, complicated culture systems remain major obstacles. These limitations are caused by the tendency of PSCs to resist differentiation by maintaining a pluripotent transcriptome network and epigenome structure (Ang et al., 2011; Azuara et al., 2006; Boyer et al., 2005). Therefore, one possible way to achieve efficient hPSC differentiation is to actively remove the epigenetic barriers from pluripotent genomes. However, this approach has not been reported in the field of stem cell differentiation.

Chromatin modifications play substantial roles in the regulation of gene expression and development (Bernstein et al., 2007; Kouzarides, 2007; Li et al., 2007). Generally, trimethylated histone H3 at lysine 4 (H3K4me3) marks transcriptionally active chromatin states, whereas trimethylated histone H3 at lysine 27 (H3K27me3) marks transcriptionally repressed chromatin states. However, in mouse and human PSCs, developmental regulatory genes are enriched in both H3K4me3 and H3K27me3, often referred to as ‘bivalent domains’ (Bernstein et al., 2006; Harikumar and Meshorer, 2015; Mikkelsen et al., 2007; Pan et al., 2007; Voigt et al., 2013; Zhao et al., 2007), where transcription is inhibited but is ‘poised’ for rapid activation when differentiation is stimulated (Voigt et al., 2013). The addition of H3K27me3 is mediated by the Polycomb-group (PcG) complex containing the core components EZH1/2, SUZ12 and EED, which suppress the expression of developmental genes in pluripotent genomes (Boyer et al., 2006; Conway et al., 2015; Lee et al., 2006; Shen et al., 2008). Removal of H3K27me3 is mediated by members of the JmjC-protein family, UTX and JMJD3 (Agger et al., 2007; Lan et al., 2007; Lee et al., 2007). UTX and JMJD3 are required for the development of various stages and cell lineages, such as mesoderm, definitive endoderm, neurons, epidermal cells, cardiac cells, M2 macrophages, and epithelial-mesenchymal transition (Burgold et al., 2008; Jiang et al., 2013; Kartikasari et al., 2013; Lee et al., 2012; Ohtani et al., 2013; Ramadoss et al., 2012; Satoh et al., 2010; Sen et al., 2008; Shpargel et al., 2014). Both UTX and JMJD3 bind to the enhancers and promoters of developmental genes in differentiated cells and de-repress transcription (Chen et al., 2012; Estaras et al., 2012; Kartikasari et al., 2013; Park et al., 2014; Tie et al., 2012).

Here, we have tested whether the catalytic activity of H3K27 demethylase can remove the epigenetic barriers from pluripotent genomes and trigger the expression of developmental regulatory genes in hPSCs. We have developed forced expression systems for H3K27 demethylase in hPSCs using synthetic, modified mRNAs or the Tet-On gene activation method. Using these gene expression systems, we have shown that the forced expression of the catalytic domain of JMJD3 contributes to the activation of tissue-specific genes mediated by lineage-defining transcription factors. We have also shown that transient expression of the catalytic domain of JMJD3 dramatically accelerates hPSC differentiation into hepatic cells or muscle cells. These results indicate the utility of H3K27 demethylase in improving the efficiency of hPSC differentiation.

## RESULTS

### Generation of H3K27me3-deficient hESCs by ectopic JMJD3 expression

**Fig. 1.**
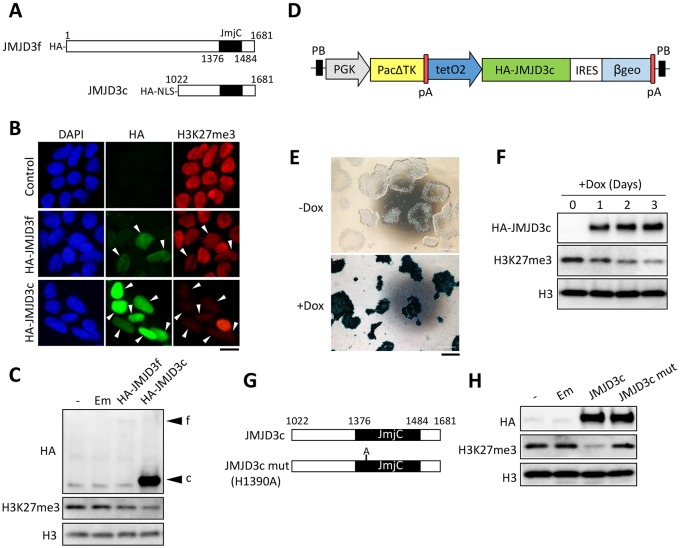
**Generation of H3K27me3-deficient hESCs by ectopic JMJD3c expression.** (A) Structures of the JMJD3f and JMJD3c proteins. JMJD3c was designed to contain the JmjC domain (amino acids 1376-1484). HA was added to JMJD3f and JMJD3c and an NLS was added to the N terminus of JMJD3c. (B) hESCs were transfected with the synthetic mRNAs for HA-JMJD3f or HA-JMJD3c and were stained with HA and H3K27me3 antibodies. Non-transfected hESCs were used as a control. The arrowheads indicate the transfected cells. The mean fluorescent intensities of H3K27me3 in the transfected cells were 0.65 for HA-JMJD3f and 0.26 for HA-JMJD3c compared with the non-transfected cells. More than 20 nuclei from three independent experiments were examined. Scale bar: 20 μm. (C) The effects of transfection of the HA-JMJD3c and HA-JMJD3f mRNAs on the H3K27me3 levels were analyzed by immunoblotting. Emerald mRNA (Em) was transfected as a control. The H3 antibody was used as a loading control. The average signal intensities of H3K27me3 from two independent biological replicates were 0.92 for Em, 0.65 for HA-JMJD3f, and 0.36 for HA-JMJD3c compared with the non-transfected cells. (D) Construct used for Tet-On induction of JMJD3c expression in hESCs (JMJD3c-hESCs). pA, polyA signal; PB, piggyBac repeat. (E) JMJD3c-hESCs were stained with X-Gal 3 days after Dox treatment. Scale bar: 500 μm. (F) HA-JMJD3c-induced H3K27me3 demethylation was detected 1 to 3 days after Dox treatment. The average signal intensities of H3K27me3 from two independent biological replicates were 0.70 on Day 1, 0.52 on Day 2, and 0.24 on Day 3 compared with Day 0. (G) A point mutation in JMJD3c (mut) was inserted at aa 1390 for catalytic inactivation. (H) Effects of HA-JMJD3c and the HA-JMJD3c mut on the H3K27me3 levels. The average signal intensities of H3K27me3 from two independent biological replicates were 0.97 for Em, 0.23 for JMJD3c, and 1.0 for the JMJD3c mut compared with the non-transfected cells.

The histone lysine demethylases (KDM1-6) possess activity against different substrates (H3K4, H3K9, H3K27 and H3K36), and their specificities have been characterized (Kooistra and Helin, 2012). UTX and JMJD3 (also known as KDM6A and KDM6B, respectively) are specific enzymes that remove di- and tri-methyl-H3K27 (H3K27me2/3). We performed a meta-analysis of previously published transcriptome data (Gifford et al., 2013) and found that among the histone demethylase genes, only JMJD3 was significantly upregulated upon hESC differentiation into the three germ layers (Fig. S1). This finding suggests that increasing levels of JMJD3 are important for inducing H3K27me3 demethylation during differentiation. Thus, we designed an experiment to overexpress JMJD3 transiently in hESCs (SEES3 line, XY) and examined whether demethylation of H3K27me3 occurs in hESCs. First, we synthesized mRNAs encoding either the full-length JMJD3 (JMJD3f) or the C-terminal region of JMJD3 containing the catalytic domain (JMJD3c) *in vitro* (Fig. 1A). The 5′ ends of these mRNAs were tagged with hemagglutinin (HA) sequences to allow us to detect the translated proteins. Eight hours after the hESCs were transfected with the synthetic mRNAs, demethylation of H3K27me3 was detected by immunostaining and immunoblotting (Fig. 1B,C). Transfection of the *JMJD3c* mRNAs induced a more significant reduction in the H3K27me3 levels than in the *JMJD3f* mRNAs, suggesting that the catalytic domain of JMJD3 is more efficient at demethylating nucleosome histones. The forced expression of the catalytic domain of another H3K27 demethylase, UTX, did not induce a significant reduction in the H3K27me3 levels in hESCs (Fig. S2A,B). Similar results were obtained using another hESC line (H9, XX; data not shown).

As an independent method of manipulating JMJD3 expression, we generated a stable hESC line (SEES3) in which HA-JMJD3c expression was controlled by doxycycline (Dox) treatment (JMJD3c-hESCs) (Fig. 1D). Dox treatment (1 μg/ml) induced HA-JMJD3c expression in essentially all hESCs (Fig. 1E), resulting in a significant reduction in H3K27me3 levels (Fig. 1F). Overexpression of the JMJD3c mutant, which lacks catalytic activity (Fig. 1G), did not induce any changes in H3K27me3 (Fig. 1H), suggesting that JMJD3c eliminates H3K27me3 through its demethylase activity.

We also confirmed that JMJD3c specifically reduced the H3K27me2/3 levels and did not significantly affect other histone methylations (H3K4me1/2/3, H3K9me1/2/3 and H3K27me1) (Fig. S2C).

### Mesoendodermal genes are upregulated in JMJD3c-hESCs

**Fig. 2.**
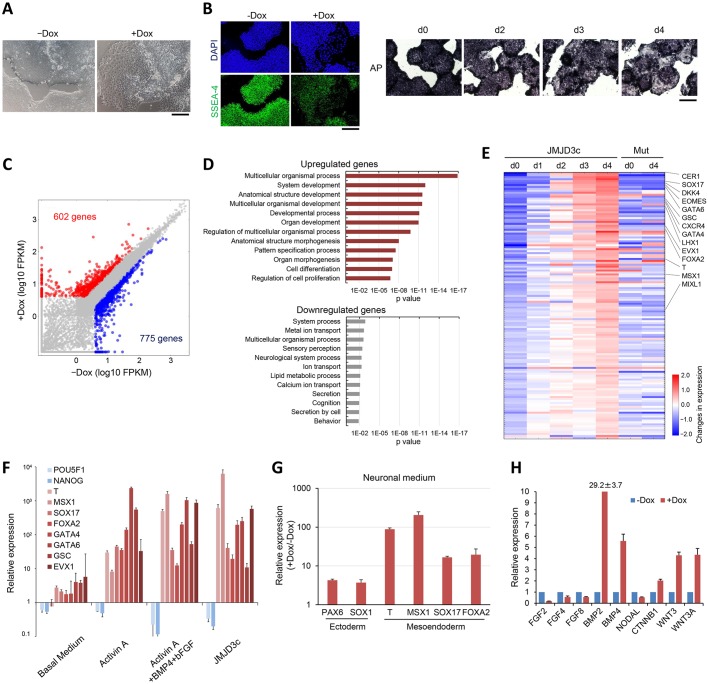
**Developmental genes are upregulated in the JMJD3c-hESCs.** (A) Morphologies of untreated (–Dox) and Dox-treated (+Dox) JMJD3c-hESCs. Scale bar: 100 μm. (B) SSEA-4 staining and alkaline phosphatase (AP) activity in the JMJD3c-hESCs after Dox treatment. Scale bars: 200 μm. (C) Scatter plot showing the comparison of the transcriptomes between the untreated (–Dox) and Dox-treated (+Dox) JMJD3c-hESCs. RNA-seq expression values (log10 of FPKM) are shown. (D) GO analyses of the up- and downregulated genes in the JMJD3c-hESCs. (E) Heat map analysis showing the changes in the expression of upregulated genes in the JMJD3c-hESCs from Day 0 to Day 4 after Dox treatment. Representative developmental genes are shown. The expression levels of these genes were not significantly changed in the JMJD3c mut-hESCs (Mut) at 4 days after Dox treatment. The color scale indicates log2 (fold change, +Dox/−Dox) gene expression. (F) qRT-PCR analyses showing the relative expression levels of pluripotent genes and mesoendodermal genes under differentiation conditions compared with hESCs. Basal medium, medium without cytokines and growth factors; Activin-A, medium used for endodermal differentiation; Activin-A+BMP4+bFGF, medium used for mesoendodermal differentiation; JMJD3c, medium with Dox. The expression levels were normalized to *GAPDH*. The error bars indicate the s.e.m. from two independent biological replicates. (G) qRT-PCR analyses showing the relative expression levels of ectodermal and mesoendodermal genes in the untreated and Dox-treated JMJD3c-hESCs cultured in neural induction medium (N2 medium). The error bars indicate the s.e.m. from three independent biological replicates. (H) qRT-PCR analyses showing the relative expression levels of FGF, BMP, NODAL and Wnt/β-catenin-related genes in the untreated and Dox-treated JMJD3c-hESCs. The error bars indicate the s.e.m. from three independent biological replicates.

We noticed that JMJD3c overexpression resulted in morphological changes in hESCs resembling differentiation (Fig. 2A), which was accompanied by reductions in the expression of the pluripotency marker SSEA-4 and alkaline phosphatase activity (Fig. 2B). The differentiating cells proliferated more slowly than the hESCs but did not exhibit increased apoptosis (Fig. S3). Differentiation was observed even under culture conditions that promote the maintenance of an undifferentiated state. To characterize the differentiation, we examined the changes in the transcriptome after JMJD3c overexpression by performing RNA-sequencing (RNA-seq) analyses of Dox-treated and untreated JMJD3c-hESCs. Among the 1377 differentially expressed genes (fold change >2, FPKM >4), 602 genes were upregulated in the Dox-treated JMJD3c-hESCs (Fig. 2C; Table S1). Gene ontology (GO) analysis revealed that these genes were enriched in development and differentiation processes (Fig. 2D). By contrast, the 775 downregulated genes were not enriched in any significant GO terms (Fig. 2D). However, as expected, pluripotency-related genes (e.g. *POU5F1*, *NANOG*, *SOX2*, *DNMT3B*, *TDGF1*, *LIN28*, *ZFP42*, *DPPA2*, *KDM4C*, *TET1*, *MYC* and *UTF1*) were downregulated in the JMJD3c-overexpressing hESCs (Fig. S4A), and more than half of these genes (*SOX2*, *DNMT3B*, *DPPA2*, *KDM4C*, *TET1*, *MYC* and *UTF1*) were included in the list of downregulated genes (fold change <0.5) (Table S2). Dox-treated JMJD3c-hESCs upregulated endogenous JMJD3 but not other KDM6 genes (Fig. S4B). The expression of the developmental genes started to increase beginning on Day 1 after Dox treatment and continued to increase (Fig. 2E). These results were accompanied by a decrease in the levels of H3K27me3 after Dox treatment (Fig. 1F). Furthermore, overexpression of the JMJD3c mutant did not induce these changes (Fig. 2E), suggesting that the H3K27me3 demethylase activity of JMJD3 is necessary and sufficient for the upregulation of developmental genes in hESCs.

More specifically, among the developmental genes that were upregulated by JMJD3c overexpression, genes related to endoderm and mesoderm differentiation, such as *SOX17*, *FOXA2*, *GATA4/6*, *EOMES*, brachyury (*T* ) and *MIXL1*, were highly expressed (Fig. 2E). To our surprise, the activation of these genes occurred in the culture conditions used to maintain an undifferentiated state. Typically, the differentiation of hESC/iPSCs into mesoderm/endoderm requires complete changes in the culture conditions using differentiation media, which contain suitable cytokines and growth factors [e.g. activin-A, bone morphogenetic protein (BMP) and fibroblast growth factor (FGF)] (Murry and Keller, 2008). Real-time PCR analyses revealed that the JMJD3c-expressing condition showed expression levels of developmental genes that were comparable to the levels observed under differentiation conditions using cytokines and growth factors (Fig. 2F). More specifically, JMJD3c-expressing hESCs showed a closer similarity to the expression patterns induced by the combination of activin-A, BMP4 and bFGF, suggesting that these cells had begun to differentiate into a mesoendodermal lineage. Interestingly, even when the JMJD3c-hESCs were cultured with Dox in neuronal induction medium, the mesoendodermal genes were strongly activated compared with the neuroectodermal genes (Fig. 2G). We noticed that JMJD3c overexpression itself upregulated *BMP2/4*, *WNT3/3A* and *CTNNB1* (β-catenin), but not FGF and NODAL genes (Fig. 2H). Transcriptome analysis also revealed that the JMJD3c-upregulated genes included BMP and Wnt signaling-related genes, such as *BMPR1B*, *FZD2* and *VANGL1* (Table S1). As BMP and Wnt/β-catenin signaling lead to hPSC differentiation towards the mesoendoderm lineage (Davidson et al., 2012; Zhang et al., 2008), ectoderm differentiation may be prevented by JMJD3c overexpression through the mesoendoderm gene regulatory network.

### JMJD3c induces genome-wide, promoter-specific H3K27 demethylation

**Fig. 3.**
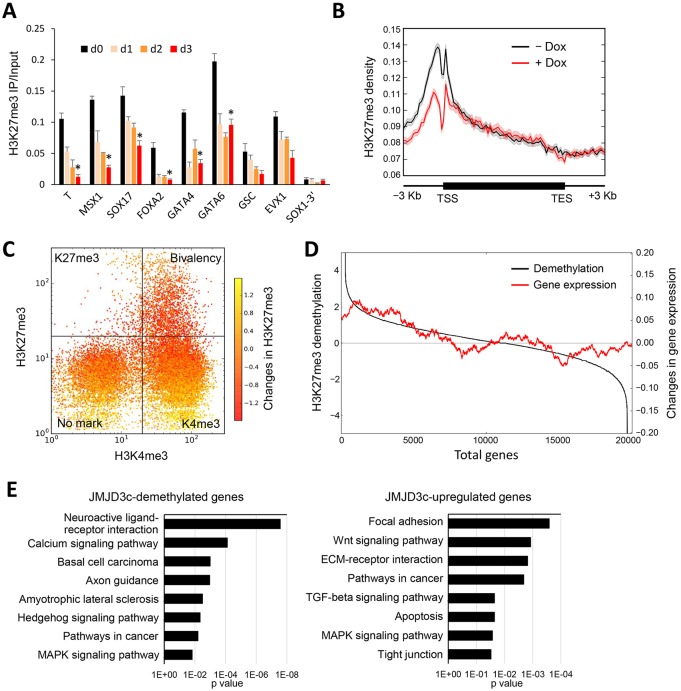
**Genome-wide reduction of H3K27me3 levels is induced by JMJD3c overexpression.** (A) Changes in the H3K27me3 levels at developmental genes in JMJD3c-hESCs resulting from Dox treatment (Days 0 to 3) were analyzed by ChIP-qPCR. The 3′ region of SOX1 (SOX1-3′) was used as a negative control. The error bars indicate the s.e.m. from three independent biological replicates. *P<0.01, *t*-test. (B) ChIP-seq analyses of H3K27me3 in JMJD3c-hESCs treated with or without Dox for 3 days. The average profiles of H3K27me3 at the genomic regions (from 3 kb upstream to 3 kb downstream of gene bodies) are shown. TES, transcription end site; TSS, transcription start site. (C) Changes in H3K27me3 levels at the promoters of 20,242 Ensembl genes. ChIP-seq analysis of H3K4me3 and H3K27me3 was performed in JMJD3c-hESCs treated with or without Dox. The plots indicate the genes categorized by the H3K4me3 and H3K27me3 levels in the pluripotent state (−Dox). K4me3, the genes marked by H3K4me3 only; K27me3, the genes marked by H3K27me3 only; Bivalency, the genes marked by H3K4me3 and H3K27me3; No mark, the genes without H3K4me3 or H3K27me3 markers. The color scale indicates the log2 (fold change) of the H3K27me3 levels in the +Dox/–Dox cells. The demethylated genes are shown in red. (D) Relationship between the changes in gene expression and H3K27me3 demethylation following JMJD3c overexpression. The total genes (20,242 Ensembl genes) were sorted according to the log2 ratio of H3K27me3 demethylation in the +Dox/–Dox cells (black). The average values of the log2 ratio of gene expression (+Dox/–Dox) in a sliding window of 1000 genes are shown (red). The upregulated genes were significantly distributed in the H3K27me3 demethylated genes (*P*-value=9.69×10^−8^). (E) Functional annotation analysis of the H3K27me3-demethylated genes and upregulated genes following JMJD3c overexpression. Nine hundred demethylated genes (fold change <0.5, +Dox/–Dox) and 603 upregulated genes (fold change >2, +Dox/–Dox) were analyzed to annotate the signaling pathways. The *P*-value indicates the significance of the GO term enrichment.

We next examined the chromatin changes in hESCs after JMJD3c overexpression. Chromatin immunoprecipitation (ChIP) analyses showed that JMJD3c overexpression resulted in a reduction in the amount of H3K27me3 at the promoters of the developmental genes (Fig. 3A). We extended the analyses to genome-wide changes in H3K27me3 by conducting ChIP-seq analyses and found that JMJD3c overexpression indeed caused a promoter-specific reduction in the H3K27me3 levels (Fig. 3B). There were no significant changes in the H3K27me3 levels at enhancer regions or repetitive elements after JMJD3c overexpression (Fig. S5A). We examined the patterns of the reduced H3K27me3 levels at the promoters marked by H3K27me3 alone and the bivalent promoters (Fig. 3C). Demethylation occurred at both of those promoters. Of the 1907 bivalent genes, 779 (41%) promoters were H3K27me3-demethylated (fold change <0.5) following JMJD3c overexpression (Table S3). When a lower threshold of demethylation levels (fold change <0.66) was used, the number of demethylated bivalent promoters increased (1186 genes, 62%). Furthermore, the reduction in the H3K27me3 levels corresponded to the upregulation of the genes (Fig. 3D). We also noted that in addition to mesoendodermal genes, neuroectodermal genes, such as *NEUROG1* and *NEUROG3*, showed demethylation of H3K27me3 (Table S3). However, these demethylations were not accompanied by upregulation of gene expression (Table S1). Indeed, JMJD3c-mediated H3K27me3-demethylated genes included many neurogenesis-related genes, whereas the JMJD3c-upregulated genes did not (Fig. 3E). On the other hand, JMJD3c significantly upregulated genes associated with the Wnt signaling pathway. These data suggest that the JMJD3c-activated mesoendodermal transcription network inhibits the expression of neuronal genes, even though the chromatin states of the neuronal genes become active. Indeed, an active marker, H3K4me3 enrichment, remained in most bivalent promoters after JMJD3c overexpression (Fig. S5B-D). Moreover, another marker of active chromatin, H3K27 acetylation (H3K27ac), did not show significant changes following JMJD3c overexpression (Fig. S5E).

### Ectopic expression of both JMJD3c and one lineage-defining transcription factor activates the expression of tissue-differentiation markers in hESCs

**Fig. 4.**
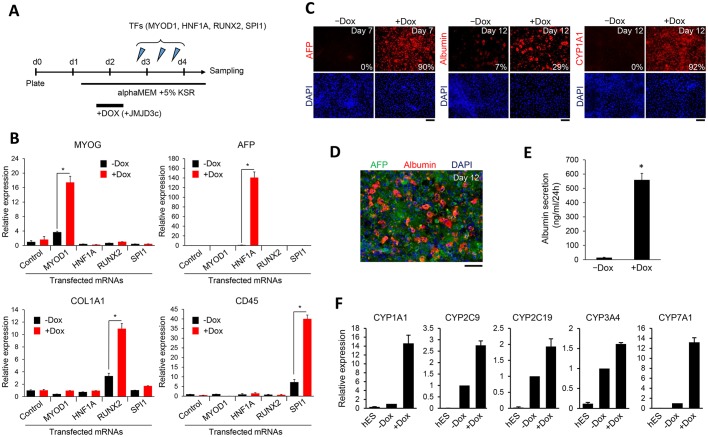
**Transient JMJD3c expression enhances hESC differentiation into multiple cell lineages.** (A) Schematic of the experimental protocol for the data shown in B. JMJD3c-hESCs were treated with or without Dox on Days 1 to 2 after plating and were transfected with synthetic mRNAs for MYOD1, HNF1A, RUNX2 or SPI1 three times on Days 2 and 3. Synthetic mRNAs for Emerald or mCherry were transfected as controls. The cells were cultured in αMEM+5% KSR (KnockOut Serum Replacement) and collected for RT-PCR analyses on Day 4. (B) qRT-PCR analyses of tissue-specific genes (*MYOG*, *AFP*, *COL1A1*, *CD45*) in transcription factor-transfected cells with (+Dox) or without (−Dox) Dox treatment. Each transcription factor indicated on the *x*-axis was transfected into the JMJD3c-hESCs. The expression levels were normalized to *GAPDH*. The error bars indicate the s.e.m. from three independent biological replicates. *P<0.05, *t*-test. (C) Hepatic differentiation of JMJD3c-hESCs. The cells were treated with or without Dox for 3 days [from Day 1 (prior to differentiation) to Day 2 (after differentiation)]. The differentiated cells were immunostained with an antibody against AFP on Day 7 and with antibodies against albumin and CYP1A1 on Day 12. Scale bars: 100 μm. The percentages of AFP-, albumin- and CYP1A1-positive cells are shown. *n*=3. (D) Co-staining of AFP and albumin in the JMJD3c-induced hepatic cells on Day 12. Scale bar: 100 μm. (E) Albumin secretion level on Day 12 after differentiation was determined by ELISA. The error bars indicate the s.e.m. from two independent biological replicates. *P<0.05, *t*-test. (F) qRT-PCR analyses of CYP450 genes in hESCs and untreated and Dox-treated hESC-derived hepatic cells. The expression levels were normalized to *GAPDH*. The error bars indicate the s.e.m. from three independent biological replicates.

Considering that repressive H3K27me3 acts as a barrier for hPSC differentiation, particularly mesoendodermal differentiation, we reasoned that the H3K27me3-deficient hESCs could be efficiently differentiated by the forced expression of lineage-defining transcription factors. We transiently overexpressed JMJD3c in hESCs and subsequently expressed one of the transcription factors MYOD1, HNF1A, RUNX2 or SPI1, which are known to regulate the differentiation of myogenic (Tapscott, 2005), hepatogenic (Si-Tayeb et al., 2010), osteogenic (Lian and Stein, 2003) or hematopoietic (Kastner and Chan, 2008) lineages, respectively, to test this hypothesis. In this procedure, we transfected the Dox-treated or untreated JMJD3c-hESCs with synthetic mRNAs encoding each transcription factor (Fig. 4A). Real-time PCR analysis revealed that when the transcription factors were overexpressed in the JMJD3c-expressing hESCs, the relevant differentiation markers [MYOG for myogenesis, AFP for hepatogenesis, COL1A1 for osteogenesis, and CD45 (PTPRC) for hematogenesis] were more significantly upregulated compared with the hESCs that did not express JMJD3c (Fig. 4B). Notably, JMJD3c alone did not change the expression patterns of these tissue-related genes. These results suggest that the combination of JMJD3c and a lineage-defining transcription factor can promote hPSC differentiation towards specific tissues or cell types.

### JMJD3c facilitates hepatocyte differentiation

As AFP expression was very significantly enhanced by JMJD3c overexpression in the above experiments, we tested the effect of JMJD3c on differentiation into hepatocytes. Hepatocytes can be generated from hPSCs in culture medium supplemented with hepatocyte growth factors (Kajiwara et al., 2012). Using this differentiation medium, we differentiated JMJD3c-hESCs into hepatocytes *in vitro*. We found that JMJD3c overexpression accelerated the timing of the expression of hepatic markers (AFP, albumin and CYP1A1) (Fig. 4C). High expression of AFP, albumin and CYP1A1 was detected in JMJD3c-overexpressing cells by Days 7 and 12 after initiating the differentiation, respectively. At these time points, the cells that did not express JMJD3c showed little to no expression of these markers. Most of the JMJD3c-overexpressing cells were differentiated into hepatic cells on Day 12, although immature hepatic cells (AFP-positive cells) were also observed (Fig. 4D). We detected albumin secretion from JMJD3c-overexpressing cells by post-differentiation Day 12 (Fig. 4E). The secretion levels (500-600 mg/ml/24 h) were comparable to that of differentiated hepatic cells after 17 days in a previous report (Kajiwara et al., 2012). Furthermore, JMJD3c-expressing hepatic cells highly expressed P450 cytochrome mRNAs (Fig. 4F). These results suggest that JMJD3c enhances the differentiation of hPSCs into mature hepatocytes.

### JMJD3c facilitates MYOD1-mediated myogenic differentiation

**Fig. 5.**
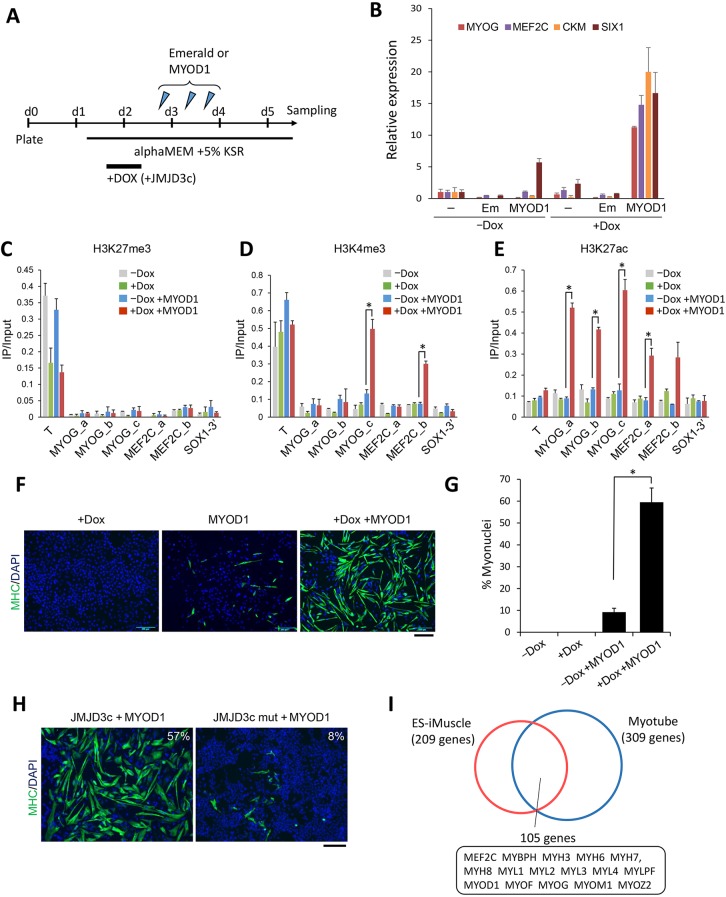
**JMJD3c facilitates the MYOD1-mediated muscle differentiation of hESCs.** (A) Schematic of the differentiation protocol. JMJD3c-hESCs were treated with or without Dox on Days 1 to 2 after plating and were transfected with synthetic mRNAs for MYOD1 or Emerald three times on Days 2 and 3. The cells were collected for each experiment on Day 5. (B) qRT-PCR analyses of the expression of myogenic genes in MYOD1-differentiated cells with (+Dox) or without (−Dox) Dox treatment. −, no transfection; Em, Emerald transfection; MYOD1, MYOD1 transfection. The expression levels were normalized to *GAPDH*. The error bars indicate the s.e.m. from three independent biological replicates. (C-E) ChIP-qPCR analyses of H3K27me3 (C), H3K4me3 (D) and H3K27ac (E) in the *MYOG* and *MEF2C* promoters of MYOD1-transfected cells with (+Dox) or without (−Dox) Dox treatment. Three (a-c) and two (a,b) regions of the *MYOG* and *MEF2C* promoters were tested, respectively. In human myoblasts, the MYOG (a,b) and MEF2C (a) regions are only enriched for H3K27ac, but not H3K4me3, whereas the MYOG (c) and MEF2C (b) regions are enriched for both markers (see also Fig. S6). T, positive control; SOX1-3′, negative control. The error bars indicate the s.e.m. from three independent biological replicates. *P<0.05, *t*-test. (F) Immunostaining for MHC in the cells overexpressing JMJD3c (+Dox), MYOD1 or both JMJD3c and MYOD1 (+Dox +MYOD1). Scale bar: 200 μm. (G) The percentage of nuclei contained within the MHC-stained cells out of total cells. The error bars indicate the s.e.m. from three independent biological replicates. *P<0.01, *t*-test. (H) MHC immunostaining in the MYOD1-transfected cells overexpressing JMJD3c or the JMJD3c mutant. The percentage of nuclei contained within the MHC-stained cells is shown. *n*=3. Scale bar: 200 μm. (I) RNA-seq analyses of upregulated genes in the myogenic cells that were induced from hESCs (ES-iMuscle) and human skeletal myotubes (Myotube). Upregulated genes were determined using ExAtlas (fold-change >4 in induced myogenic cells and >10 in myotubes relative to hESCs, z-value >4).

We found that JMJD3c overexpression activated the *PAX3* and *PAX7* genes (Fig. S6), both of which are markers for skeletal muscle progenitors (Buckingham and Relaix, 2007). This finding prompted us to examine whether JMJD3c can accelerate MYOD1-induced muscle differentiation. It is known that forced expression of MYOD1 alone cannot cause sufficient epigenetic and transcriptional changes in hPSCs, resulting in poor myogenic conversion of hPSCs (Albini et al., 2013). To examine whether JMJD3c accelerates MYOD1-mediated myogenic differentiation, we transiently overexpressed JMJD3c in hESCs before MYOD1 overexpression (Fig. 5A). We examined the changes in the expression of markers for skeletal muscle differentiation – *MYOG*, *MEF2C*, *CKM* and *SIX1* (Faralli and Dilworth, 2012). Real-time PCR analyses revealed that MYOD1 overexpression alone could not induce the upregulation of myogenic genes, except for *SIX1* (Fig. 5B), an early marker of myogenesis (Relaix et al., 2013). However, when JMJD3c was overexpressed prior to MYOD1 overexpression, all of these genes showed significant upregulation.

We also investigated the chromatin changes in the promoter regions of *MYOG* and *MEF2C* during MYOD1-mediated differentiation with or without JMJD3c overexpression using the ChIP assay. We examined the promoter regions that are known to exhibit enrichment of active chromatin markers (H3K4me3 and H3K27ac) in human primary myoblasts (Fig. S7). In myoblasts, all of the regions were enriched for H3K27ac, and two regions were enriched for both H3K4me3 and H3K27ac (Fig. S6). The levels of H3K27me3 in all of those regions were lower than those of a positive control gene, brachyury (*T*), in both hESCs and differentiated cells (Fig. 5C). However, as we expected, H3K4me3 was significantly upregulated at two regions in the JMJD3c-overexpressing cells (Fig. 5D). We also found that all of the regions were significantly enriched for H3K27ac in the MYOD1-overexpressing cells following JMJD3c overexpression, but not in the absence of JMJD3c overexpression (Fig. 5E). Overexpression of JMJD3c alone (without MYOD1 overexpression) did not induce H3K27ac enrichment in the myogenic promoters (Fig. 5E), suggesting that the combination of JMJD3c and MYOD1 is required for the generation of active chromatin of myogenic genes.

At 4 days post differentiation, JMJD3c/MYOD1-overexpressing hESCs expressed markers of mature muscles: myosin heavy chain (MHC) and myotube-like morphology (Fig. 5F). The percentage of MHC-positive cells was much higher in the JMJD3c/MYOD1-overexpressing hESCs compared with the MYOD1-overexpressing hESCs (Fig. 5G). Notably, forced expression of the JMJD3c mutant and UTX did not induce MYOD1-mediated myogenic differentiation (Fig. 5H; Fig. S8), suggesting that JMJD3c specifically functions as a H3K27 demethylase for the myogenic differentiation of hPSCs.

To characterize the muscle cells generated by overexpressing both JMJD3c and MYOD1, we compared the gene expression patterns of these cells with the expression patterns of human skeletal myotubes. Half of the genes (105 genes) that were upregulated in the generated muscle cells were highly expressed in skeletal myotubes, including genes important for mature muscle cells (Fig. 5I; Table S4). We also found that further differentiation of the generated muscle cells produced myotube-like cells that exhibited spontaneous contractions (Movie 1).

### Differentiation of hPSCs into skeletal muscles using synthetic mRNAs alone

**Fig. 6.**
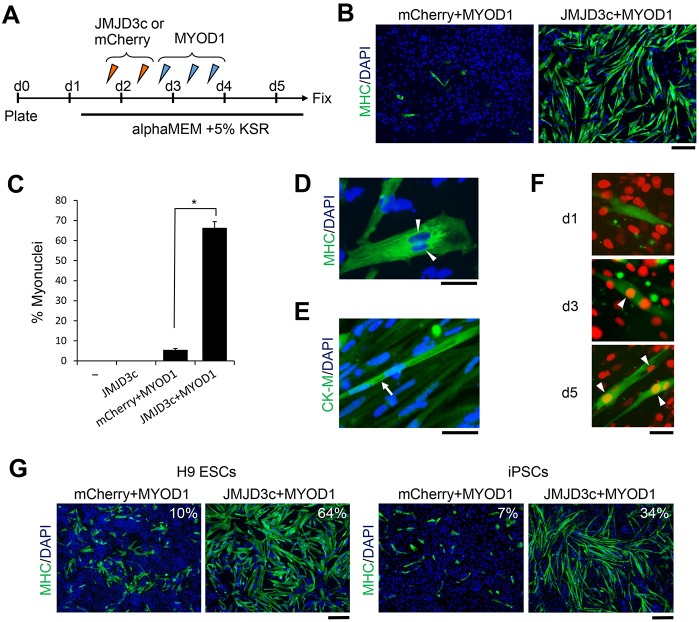
**Synthetic mRNA-mediated myogenic differentiation of hESCs and iPSCs.** (A) Schematic of the differentiation protocol. hESC/iPSCs were transfected with synthetic mRNAs for JMJD3c or mCherry twice on Days 1 and 2 and MYOD1 three times on Days 2 and 3. The cells were fixed for immunostaining on Day 5. (B) MHC immunostaining in cells that were transfected with MYOD1 after mCherry or JMJD3c transfection. Scale bar: 200 μm. (C) The percentage of nuclei contained within MHC-stained cells. The error bars indicate the s.e.m. from three independent biological replicates. *P<0.01, *t*-test. −, no transfection. (D) Representative image showing muscular fusion (arrowheads). Scale bar: 50 μm. (E) Immunostaining for creatine kinase M (CK-M). The arrow shows a mature skeletal muscle. Scale bar: 50 μm. (F) Induced myogenic cells were labeled with green fluorescence and co-cultured with mouse C2C12 cells, nuclei of which were labeled with red fluorescence. On Days 3 and 5 after co-culturing, cell fusions were detected (arrowheads). Scale bar: 50 μm. (G) H9-hESCs and hiPSCs derived from BJ fibroblasts were transfected with mCherry or JMJD3c, followed by MYOD1, and were immunostained for MHC. Scale bar: 200 μm. *n*=3. The percentage of nuclei contained within MHC-stained cells is shown.

Finally, to develop a footprint-free system, we attempted to differentiate hESCs into skeletal muscle cells using only synthetic mRNAs for both JMJD3c and MYOD1. The synthetic mRNAs for JMJD3c were transfected into the hESCs twice, followed by three transfections with the MYOD1 synthetic mRNAs (Fig. 6A). Two days after the last transfection of the MYOD1 mRNAs, the majority of hESCs were differentiated into MHC-positive cells (Fig. 6B,C). As a control, we transfected hESCs with synthetic mRNAs for mCherry and MYOD1, which failed to induce myogenic differentiation. Importantly, some MHC-positive cells appeared to be fused cells (Fig. 6D) in which a mature myogenic marker, creatine kinase-M, was highly expressed (Fig. 6E). The fusion capability was confirmed by a cell fusion assay with mouse C2C12 myoblast cells (Fig. 6F). These results suggest that the myotube-like cells induced by the JMJD3c and MYOD1 synthetic mRNAs have the potential to become mature skeletal muscles *in vitro*. JMJD3c also accelerated MYOD1-mediated differentiation of H9 hESCs and hiPSCs (Fig. 6G), suggesting that JMJD3c generally facilitates the direct conversion of pluripotent cells into terminally differentiated cells.

## DISCUSSION

Although it has been recognized that epigenetic mechanisms play important roles in the transcriptomic changes observed during differentiation (Gifford et al., 2013; Xie et al., 2013), the epigenetic factors that are primarily responsible for establishing a differentiated state are currently unknown. Previous studies using JMJD3 knockout or knockdown have shown that JMJD3 is required for the activation of mammalian developmental programs (Burgold et al., 2008; Jiang et al., 2013; Ohtani et al., 2013; Satoh et al., 2010; Sen et al., 2008). It has also been shown that JMJD3 functions as an epigenetic barrier during the reprogramming of differentiated cells into pluripotent stem cells (Zhao et al., 2013). In this study, we have revealed that the catalytic domain of histone demethylase JMJD3 (JMJD3c) can drive the activation of developmental genes in hESCs in the absence of environmental changes. Moreover, JMJD3c facilitates switching of the hESC gene expression patterns to the patterns of terminally differentiated cells. These results suggest that JMJD3 is a crucial determinant of the differences in the chromatin characteristics between undifferentiated and differentiated mammalian cells.

Forced expression of JMJD3c enabled the hESCs to exit from the pluripotent state and to upregulate developmental genes that are associated with H3K27me3 demethylation. The catalytic mutant of JMJD3c failed to induce these phenomena, suggesting that H3K27 demethylation by JMJD3c is directly involved in the transcriptional activity of developmental genes. Notably, overexpression of the JmjC domain of UTX (66% sequence homology with JMJD3c) failed to induce H3K27me3 demethylation and hESC differentiation, suggesting that there are functional differences between JMJD3 and UTX. Some reports revealed that UTX regulates gene expression during ESC differentiation independently of demethylase activity (Morales Torres et al., 2013; Wang et al., 2012), indicating that UTX is involved in different types of transcriptional machinery compared with JMJD3. Furthermore, in contrast to JMJD3, UTX has positive functions in iPSC reprogramming (Mansour et al., 2012), suggesting that UTX is involved in the establishment of the methylation pattern in the pluripotent state. UTX may function to maintain H3K27 demethylation at genes other than bivalent genes.

Previous reports revealed that JMJD3 regulates the Wnt signaling pathway and interacts with β-catenin to activate target genes during the mesoendoderm differentiation of ESCs (Jiang et al., 2013; Ohtani et al., 2013). In fact, JMJD3c overexpression upregulated Wnt and BMP signaling genes in our system. Moreover, a recent study reported that upon differentiation, *Jmjd3* knockout ESCs showed significant repression of genes involved in mesoendoderm development, but the ectodermal markers were not affected (Ohtani et al., 2013). However, another group revealed that JMJD3 controls the expression of neuroectodermal genes during neural ESC differentiation (Burgold et al., 2008). JMJD3 also interacts with SMAD3 to activate neural markers in neural stem cells after TGFβ stimulation (Estaras et al., 2012). These findings suggest that JMJD3 potentially recruits the regulatory regions of every target gene. In this study, JMJD3c overexpression preferentially induced the expression of mesoendodermal genes, although H3K27me3 was demethylated genome-wide, including the promoters of ectodermal genes. From these results, we have hypothesized the following mechanism: JMJD3c could target and demethylate H3K27me3 on the promoters of any genes related to three germ layers. Among those genes, the key genes associated with Wnt and BMP signal pathway are activated, which preferentially generates the transcriptional network for meso/endodermal induction and inhibits ectoderm differentiation.

The PcG components EZH2, EED and SUZ12 are present at the promoters of key developmental genes in the pluripotent state to repress transcription (Boyer et al., 2006; Ku et al., 2008; Lee et al., 2006). Mouse ESCs lacking *Suz12* exhibit global loss of H3K27me3 and de-repression of developmental genes (Pasini et al., 2007), which are the same phenotypes exhibited by the Dox-treated JMJD3c-hESCs. However, the *Suz12*-null ESCs fail to activate developmental genes when the cells are induced to differentiate (Pasini et al., 2007), suggesting that PcG proteins are required for the upregulation of developmental genes during hESC differentiation. JMJD3 might cooperatively function with PcG proteins to modulate the expression of mesoendodermal genes after hESC differentiation.

We have shown that JMJD3c overexpression followed by MYOD1 overexpression significantly increases the efficiency of myogenic differentiation of hPSCs. The myogenic gene program is quickly activated through epigenetic changes only when JMJD3c and MYOD1 are sequentially expressed in hESCs. Interestingly, because H3K27me3 is not enriched at the promoters of myogenic genes (*MYOG* and *MEF2C*) in hESCs, the demethylase activity of JMJD3c seems to be indirectly involved in the activation of myogenic genes. The JMJD3c-upregulated mesodermal developmental genes may include the transcriptional co-regulators of MYOD1, enabling the effective activation of myogenic gene expression. Indeed, JMJD3c alone induces the expression of the early myogenic regulators PAX3 and PAX7 in addition to mesodermal differentiation, which could facilitate myogenic terminal differentiation. Alternatively, JMJD3c might cooperate with MYOD1 to activate the myogenic genes by changing the chromatin structures of their regulatory regions. Indeed, when JMJD3c is expressed prior to MYOD1, H3K4me3 and H3K27ac are upregulated at the *MYOG* and *MEF2C* promoters. These results suggest that JMJD3c plays key roles in the alteration of the chromatin structures of hPSCs, not only for early differentiation, such as for mesoendodermal differentiation, but also for differentiation into tissue-specific lineages. The SWI/SNF chromatin remodeling component BAF60C (SMARCD3) also promotes MYOD-mediated myogenic conversion in hESCs (Albini et al., 2013), supporting the idea that remodeling of the chromatin structure is important for the transcription factor-mediated conversion of pluripotent stem cells into terminally differentiated cells. The detailed mechanisms that promote the ‘directed differentiation’ of hPSCs through epigenetic regulation should be elucidated in the future.

Previous studies have shown that the forced expression of the transcription factors MYOD1 or PAX7 can generate differentiated myogenic cells from hPSCs *in vitro* (Darabi et al., 2012; Goudenege et al., 2012; Tedesco et al., 2012). In these experiments, the transcription factors were transduced using lentiviral or adenoviral vectors, and those factors were overexpressed after mesodermal or mesenchymal cells were produced from hPSCs. These procedures require multiple differentiation steps and several weeks for complete myogenic differentiation. A recent report showed that when a *piggyBac* vector system was used to express MYOD1, the transgenic hiPSCs could be directly converted into skeletal muscle cells at high efficiency (Tanaka et al., 2013), suggesting that an extremely large amount of MYOD1 is required to activate the myogenic program in PSCs. Indeed, MYOD1-mediated muscle differentiation from hESCs has low reproducibility compared with muscle differentiation from fibroblasts (Albini et al., 2013). In this study, we have reported the efficient (>60%) and rapid (4 days) differentiation of hPSCs with synthetic mRNAs encoding JMJD3c and MYOD1. Because RNA-based protein expression is transient and has no risk of genomic integration and mutagenesis, this approach may be suitable for cell-based therapies in regenerative medicine.

Because JMJD3c overexpression can initiate the transcriptome program for mesoendodermal lineages, it is conceivable that our method, i.e. the forced expression of JMJD3c and tissue-specific transcription factor(s), can also be applied to differentiation towards specific tissue and cell types other than muscle cells. Indeed, here, we showed that the transient expression of JMJD3c and subsequent expression of HNF1A, RUNX2 or SPI1 activates the differentiation markers for the hepatoblast, osteoblast or hematopoietic lineages, respectively. Moreover, JMJD3c overexpression enhanced hepatic differentiation achieved with an established protocol. These results suggest that our system has broad utility for various cellular lineages.

Although the differentiation potential of hiPSCs holds great promise for therapeutic applications, there are significant variations in the differentiation capacities of hiPSC lines (Boulting et al., 2011), which will limit the utility of iPSCs in regenerative medicine. These variations are thought to be caused by epigenetic differences (Bock et al., 2011; Lister et al., 2011; Ohi et al., 2011). Our results showed less efficient myogenic differentiation in a hiPSC line than that in hESCs, which might be due to the epigenetic differences, including DNA methylation and histone modifications, between these cell types. Based on these results, we propose that manipulating H3K27me3 or other modifications with our method allows the alteration of the epigenetic patterns of low-potential iPSC lines and improves their differentiation capacities.

## MATERIALS AND METHODS

### hPSC culture

SEES-3 hESCs (Akutsu et al., 2015) were obtained from the Center for Regenerative Medicine, National Research Institute for Child Health and Development, Japan. H9 hESCs were obtained from WiCell Research Institute, USA. The hiPSCs were generated from adult human BJ fibroblasts (male) by transfecting them with *POU5F1*, *SOX2*, *KLF4* and *MYC* mRNAs. The hESCs/iPSCs were maintained in feeder-free conditions using StemFit AK-03 medium (Ajinomoto) on iMatrix-511 (Nippi)-coated plates. The culture conditions for differentiation are described in the supplementary Materials and Methods section. All experiments were performed in accordance with the Guidelines for Derivation and Utilization of Human Embryonic Stem Cells of the Ministry of Education, Culture, Sports, Science, and Technology, Japan.

### Generation of inducible JMJD3c-hESC lines

A full-length human JMJD3 clone was obtained from Addgene (plasmid ID #24167). The point mutation in the catalytic domain was inserted using a PrimeSTAR Mutagenesis Basal Kit (Takara). The HA-tagged JMJD3c and mutant sequences, to which nuclear localization signal (NLS) sequences were added, were subcloned into a *piggyBac* construct containing the Tet-responsive element, IRES-βgeo, and PGK promoter controlling the puromycin resistance gene. SEES-3 hESCs that consistently expressed the reverse Tet-transactivator were co-transfected with these vectors and *piggyBac* transposase vectors using the GeneJuice transfection reagent (Novagen). Stable clones were generated by puromycin selection. Inducible expression upon Dox treatment was confirmed by X-Gal staining.

### Modified RNA synthesis and transfection

The modified mRNAs were synthesized as previously described (Warren et al., 2010). RNA transfections were performed with Lipofectamine 2000 (Invitrogen) or Lipofectamine Messenger Max (Invitrogen), according to the manufacturer's instructions. For further details, see supplementary Materials and Methods.

### Immunostaining and immunoblotting

Antibodies used in this study are listed in Table S5.

For immunostaining, the cells were fixed in 4% paraformaldehyde (PFA) for 10 minutes at room temperature (RT) and permeabilized in 0.5% Triton X-100 in PBS for 10 minutes. The cells were blocked in PBS and 2% bovine serum albumin (BSA) for 10 minutes and incubated with the primary antibodies in a blocking solution (1:500) for 2-3 h at RT or overnight at 4°C. After two washes in PBS, the cells were incubated with Alexa-conjugated secondary antibodies (Invitrogen) in a blocking solution (1:500) for 1 h at RT. Nuclei were counterstained with DAPI (Dako) for 5 min at RT. Immunofluorescence was visualized with an inverted fluorescence microscope IX73 (Olympus). Images were obtained using Olympus cellSens imaging software. The fluorescence intensities were quantified using ImageJ software. More than 20 nuclei from three independent experiments were examined to calculate the average intensities.

For immunoblotting, the cells were lysed with a sample buffer (50 mM Tris-HCl, pH 6.8, 2% SDS, 6% 2-mercaptoethanol and 500 mg/ml urea). The proteins were separated by SDS-PAGE on a 4-15% polyacrylamide gel (Bio-Rad) and were electrically transferred to polyvinylidene difluoride membranes (Bio-Rad). The membranes were blocked for 1 h in Tris-buffered saline containing 0.1% Tween-20 (TBST) and 5% skimmed milk. The membranes were washed in TBST and then incubated with primary antibodies in TBS/2% BSA (1:1000) overnight at 4°C. The membranes were washed and then incubated with horseradish peroxidase-conjugated secondary antibodies (GE Healthcare) (1:2000) for 1 h at RT. The membranes were washed in TBST, and immunoreactivity was visualized using an ECL Prime Detection Kit (GE Healthcare) and detected using a Luminescent Image Analyzer (LAS-4000; Fujifilm). Immunoblots were quantified using ImageJ software. The signal intensity levels were normalized to the loading controls, and the average values were calculated from two or three independent experiments.

### Alkaline phosphatase staining

Alkaline phosphatase activity was visualized with the BCIP/NBT Substrate System (Dako).

### Apoptosis assay

Apoptosis was detected using the Annexin V-FITC Apoptosis Detection kit (MBL) and analyzed by flow cytometry (FACSAria II, BD Biosciences).

### qRT-PCR

Total RNA was isolated with TRIzol reagent (Invitrogen), and cDNAs were generated with random hexamers using the Superscript III First-strand Synthesis Kit (Invitrogen) or the ReverTra Ace kit (Toyobo). Real-time PCR was performed using a SYBR Green PCR system (Takara). The primer sequences used for RT-PCR are listed in Table S6.

### cDNA library preparation

Total RNA was extracted using TRIzol solution (Roche) according to the manufacturer's instructions. The cDNA library was prepared from 500 ng of each total RNA sample for massive parallel sequencing using an NEBNext Poly(A) mRNA Magnetic Isolation Module and an Ultra Directional RNA Library Prep Kit for Illumina (NEB). The cDNA library produced ranged from 400 to 1000 bp, including the adaptor sequences.

### RNA-seq analysis

RNA-seq was performed with an Illumina MiSeq for 150 single-ended base pairs. The sequence reads were mapped to the human genome (hg19) using TopHat v2.0.13. The expression values for genes were calculated as fragments per kilobase of exon per million mapped reads (FPKM) using Cufflinks v2.1.1. The functional annotation of the genes was performed using DAVID software. Some of the comparisons of gene expression were performed with ExAtlas software (Sharov et al., 2015).

### ChIP analysis

The ChIP experiment was performed as previously described (Akiyama et al., 2015). The primer sequences are listed in Table S6.

### ChIP-seq analysis

The ChIPed DNA libraries were constructed using an NEBNext ChIP-Seq Library Prep Kit for Illumina (NEB) and were sequenced on an Illumina MiSeq for 150 single-ended base pairs. The sequence reads were mapped to the human genome (hg19) using Bowtie2 (Langmead and Salzberg, 2012). The reads that mapped to the mitochondrial genome were excluded from the subsequent analyses. The chromatin profiles of H3K27me3 and H3K4me3 at the genomic regions were generated with ngs.plot (Shen et al., 2014). The profiles were normalized to the average density of methylation from the transcription end site to +6 kb. Bivalent genes were defined as genes that were enriched both in H3K4me3 and H3K27me3 (ngs.plot_hm values >20 in ±3 kb from the transcription start site). Seventy percent of the bivalent genes defined here overlapped with the bivalent genes defined in previous reports (Pan et al., 2007; Zhao et al., 2007). The Ensembl genes were sorted based on the fold changes of the H3K27me3 occupancy (±3 kb from the transcription start site) in the JMJD3c-hESCs that were treated with or without Dox to construct the rank-plot. The expression changes were estimated in a sliding window of 1000 genes.

### Albumin secretion assay

The concentration of albumin secreted into the culture media by the differentiated cells was determined using a Human Albumin ELISA Quantitation Kit (Bethyl) according to the manufacturer's instructions.

### Analyses of the induced myogenic cells

The nuclei contained within the MHC-stained cells were counted to estimate the number of myogenic cells that differentiated from the hPSCs after 4 days. As differentiated cells with multiple nuclei represent a small population (< 5%) at this point, the cells with multiple nuclei were counted as one myonucleus. For the cell fusion assay, the induced myogenic cells were labeled with green fluorescence after transfection with Emerald mRNA. The cells were co-cultured with C2C12 cells expressing H2B-mCherry in medium consisting of DMEM (Gibco) supplemented with 2% horse serum. Human myoblasts, HSMM cells (Lonza), were cultured in DMEM-F12 (Gibco) supplemented with 2% horse serum for 5 days to generate skeletal myotubes.

### Statistical analysis

The statistical significance of the differences between samples was assessed using Student's *t-*test for independent samples. In the sequencing analyses, the correlation between H3K27me3 demethylation and gene upregulation was assessed using PAGE analyses (Kim and Volsky, 2005).
